# *Mi-1.2*, an R gene for aphid resistance in tomato, has direct negative effects on a zoophytophagous biocontrol agent, *Orius insidiosus*

**DOI:** 10.1093/jxb/eru361

**Published:** 2014-09-04

**Authors:** Godshen R. Pallipparambil, Ronald J. Sayler, Jeffrey P. Shapiro, Jean M. G. Thomas, Timothy J. Kring, Fiona L. Goggin

**Affiliations:** ^1^319 Agricultural Building, Department of Entomology, University of Arkansas, Fayetteville, AR 72701, USA; ^2^Plant Science Building, Department of Plant Pathology, University of Arkansas, Fayetteville, AR 72701, USA; ^3^USDA, ARS, CMAVE, 1700 SW 23^rd^ Dr., Gainesville, FL 32608-1069, USA

**Keywords:** Biological control, host plant resistance, insect resistance, integrated pest management, *Mi-1*, *Mi-1.2*, mesophyll feeding, natural enemy, plant defence, R gene, xylem feeding.

## Abstract

*Mi-1.2* is a single dominant gene in tomato that confers race-specific resistance against certain phloem-feeding herbivores including aphids, whiteflies, psyllids, and root-knot nematodes. Few prior studies have considered the potential non-target effects of race-specific resistance genes (R genes), and this paper evaluates the compatibility of *Mi*-mediated resistance in tomato with a beneficial zoophytophagous predator, *Orius insidiosus* (Say). In addition to preying on aphids and other pests, this piercing–sucking insect also feeds from the xylem, epidermis, and/or mesophyll, and oviposits within plant tissues. Comparison of *O. insidiosus* confined to isogenic tomato plants with and without *Mi-1.2* revealed that immatures of *O. insidiosus* had lower survival on resistant plants even when the immatures were provisioned with prey that did not feed on the host plant. Molecular gut content analysis confirmed that adults and immatures of *O. insidiosus* feed on both resistant (*Mi-1.2*+) and susceptible (*Mi-1.2–*) genotypes, and bioassays suggest that resistance does not affect oviposition rates, plant sampling, or prey acceptance by *O. insidiosus* adults. These results demonstrate a direct negative impact of R-gene-mediated host plant resistance on a non-target beneficial species, and reveal that *Mi*-mediated resistance can impact organisms that do not feed on phloem sap. Through laser capture microdissection and RT-PCR, *Mi-1.2* transcripts were detected in the epidermis and mesophyll as well as the phloem of tomato plants, consistent with our observations that *Mi*-mediated resistance is active outside the phloem. These results suggest that the mode of action and potential ecological impacts of *Mi*-mediated resistance are broader than previously assumed.

## Summary

This paper reports that even specific targeted forms of pest resistance in plants can have non-target effects, and may not always be compatible with biological control for integrated pest management.

## Introduction

Although insect-resistant crop varieties and biological control agents are both important components of integrated pest management, they are not always fully compatible, because many of the resistance mechanisms that protect plants from herbivorous insects may also deter beneficial predatory insects. For example, structural defences such as trichomes, epidermal thickness, and waxy surfaces can impede prey-finding by predators (Obrycki, 1986; Eigenbrode, 2001; Kennedy, 2003), and certain secondary metabolites that deter herbivory may also have toxic effects on herbivores’ natural enemies (Ode, 2006). Therefore, to assess the costs, benefits, and ecological impacts of host plant resistance, it is critical to examine the effects of resistance on biological control agents. Specifically, it must be determined whether the resistance factors are biologically active against natural enemies and whether natural enemies encounter these factors while foraging for prey.

Most studies on the effects of host plant resistance on biological control agents have focused on fairly broad-spectrum defences that persist over time and are expressed throughout the plant, such as trichomes or insecticidal transgenes (Ode, 2006; Gatehouse *et al.*, 2011). By contrast, fewer studies have examined the potential non-target impacts of monogenic resistance genes (R genes) in plants. These genes typically confer resistance against one or a small number of pest species, and may not be effective against all biotypes within the target species. Each R gene enables the host plant to recognize the presence of a specific gene product from avirulent biotypes of the pest which, in turn, triggers a rapid localized defence response at the site of attack (van Ooijen *et al.*, 2007). Although the concept of R genes was first defined to describe plant–pathogen interactions (Flor, 1955), it has since been found to apply to many sources of resistance against insects, including the Hessian fly, aphids, and planthoppers (Smith and Clement, 2012). Because R-gene-mediated resistance relies on highly specific recognition events and on downstream defences that are induced rather than pre-formed, transient rather than persistent, and often local rather than systemic, non-target organisms are expected to have limited exposure to these defences. However, several recent studies indicate that R genes can nonetheless have non-target effects (Lundgren *et al.*, 2009*b*;Mitchell *et al.*, 2010; Chacón *et al.*, 2012; Ghising *et al.*, 2012). Moreover, certain R genes have been demonstrated to respond to unrelated effectors from taxonomically divergent organisms (Lozano-Torres *et al.*, 2012) and, to date, there are no accurate methods to predict the specificity of R genes based on nucleotide sequences or taxonomic relationships. Therefore, it is important to test the target range of R gene-mediated resistance empirically.

The *Mi-1.2* herbivore resistance gene in tomato (*Solanum lycopersicum* L.) is a suitable system to study the specificity and potential non-target effects of R gene-mediated resistance because it affects a larger and more diverse set of pest species than any other known R gene. This gene, which is present in numerous commercial tomato cultivars, confers resistance against certain biotypes of the potato aphid *Macrosiphum euphorbiae* (Thomas) (Rossi *et al.*, 1998), the sweetpotato whitefly *Bemisia tabaci* (Gennadius) (Nombela *et al.*, 2001), the tomato psyllid *Bactericerca cockerelli* (Sulc) (Casteel *et al.*, 2006), and certain species of root-knot nematode, *Meloidogyne* spp. (Milligan *et al.*, 1998). All of these pests are piercing-sucking herbivores that feed on phloem sap and previous studies with aphids and nematodes indicate that resistance acts primarily by blocking the establishment of a feeding site in the vascular tissue of the plant (Dropkin, 1969; Kaloshian *et al.*, 2000). These observations suggest that the effects of *Mi-1.2* may be limited to phloem-feeding organisms; however, more recent studies of whitefly and aphid feeding behaviour on resistant plants indicate that *Mi*-mediated resistance also involves deterrent factors that herbivores encounter even before contacting the phloem (Jiang *et al.*, 2001; Pallipparambil *et al.*, 2010). These findings open the possibility that insect feeding guilds other than phloem sap-feeding herbivores could encounter *Mi*-mediated defences.

The potential non-target effects of the *Mi-1.2* resistance gene in tomato on a zoophytophagous generalist predator, the minute pirate bug [*Orius insidiosus* (Say)] have been investigated here. Zoophytophagous predators have supplementary plant feeding behaviour, and are particularly important in providing consistent pest suppression because they are less likely to disperse or starve when herbivores are scarce (Eubanks and Styrsky, 2005). However, they may also be more vulnerable than strict predators to adverse effects of plant defences because of increased direct exposure. *Orius insidiosus* oviposit into leaf tissues and their preference for oviposition sites is influenced by surface characteristics of the host plant (Lundgren *et al.*, 2008). Moreover, this species feeds on pollen, xylem sap, and the contents of epidermal and mesophyll cells in addition to preying on aphids and other small arthropods (Armer *et al.*, 1998). Early instars of *O. insidiosus* rely heavily on plant feeding to meet their nutritional needs (Coll, 1996), and certain studies suggest that plant suitability for early instars may override prey availability for the predator. Therefore, *O. insidiosus* is a good species to use for initial risk assessments because it has high exposure levels to the host plant. Moreover, it is a common, commercially available biological control agent and a very influential species in food webs. *Orius insidiosus* feed on predaceous/herbivorous thrips, mites, aphids, and insect eggs and, in turn, fall prey to other predaceous arthropods including spiders, green lacewings, and other Hemipterans. Thus, this species can have a large impact on multitrophic population dynamics in agroecosystems.

To understand the potential effects of *Mi*-mediated resistance on this critical predator species, adult feeding behaviour, oviposition, nymphal emergence and survival on isogenic tomato lines with and without *Mi-1.2*, as well as their acceptance of prey items (aphids) reared on these genotypes were compared. Because it is critical to confirm that these predators interact directly with the plant tissues in which resistance is expressed, a combination of laser-capture microdissection and RT-PCR was also used to identify the tissue types in which *Mi-1.2* is transcribed, and real-time PCR was performed to confirm the incidence of *O. insidiosus* feeding on plants with (*Mi-1.2+*) and without (*Mi-1.2–*) this resistance gene. Our broad objectives were to investigate the compatibility of a widely deployed R gene with a common biological control agent, and to shed light on the potential ecological impacts of R gene-mediated resistance.

## Materials and methods

### Plants and insects

This study used near-isogenic tomato cultivars with (cv. Motelle) and without (cv. Moneymaker) a ~650kb region carrying the *Mi-1.2* resistance gene, and a transgenic line (143-25) that expresses *Mi-1.2* driven by its native promoter in a Moneymaker background (Milligan *et al.*, 1998). Tomato plants were used for experimentation at flowering, ~5 weeks after germination. *Orius insidiosus* were cultured in humidified plastic chambers (~24 °C, 16:8h light:dark) with Hydrocapsules® (Analytical Research Systems, Gainesville, FL) as a water source, *Ephestia kuehniella* Zeller eggs (Beneficial Insectary, Oak Run, CA) as a prey source, and green beans for oviposition and plant feeding. This study used a clonal population of the potato aphid (clone WU12) that can survive and reproduce on plants with *Mi-1.2*, although its population growth is greater on susceptible cultivars (Goggin *et al.*, 2001, 2004; Cooper *et al.*, 2004).

### *Orius insidiosus* oviposition, emergence, and survival

Oviposition-deprived mated female *O. insidiosus* were enclosed in clip cages (5cm diameter) with moth eggs at uniform leaf positions on Moneymaker (*Mi-1.2–*), Motelle (*Mi-1.2+*), and 143-25 (*Mi-1.2+*) plants maintained in growth chambers (85% RH, 24 °C, 16:8h light:dark; 1 female per cage; 3 cages per plant; 10 plants per genotype). Adults were removed after oviposition (60h), and cages were observed daily to record the number of immatures that emerged. After all of the live insects reached the second instar, early-instar mortality was recorded and immatures were caged individually to prevent cannibalism (3–4 cages per plant, 1 immature per cage). Moth eggs were removed and cages were subsequently monitored daily until the insects molted to adulthood or died in order to calculate total mortality. The number of eggs laid by the original females during their 60h oviposition period was also determined at the end of the assay by detaching the leaves and observing them under a light microscope for hatched and unhatched eggs. Emergence was calculated for each cage by dividing the number of immatures observed by the number of eggs embedded in the leaf. The number of eggs laid, per cent emergence, per cent early-instar mortality, and per cent total mortality were analysed by ANOVA using JMP v9 (SAS Institute, Cary, NC, USA).

### *Orius insidiosus* feeding behaviour

Choice and no-choice assays were used to determine whether *Mi-1.2* influences *O. insidiosus* feeding behaviour on either plants or aphids. Adult females of *O. insidiosus* were deprived of plant materials for ~15h and were then caged individually in a transparent 0.2ml tube with either a resistant (Motelle, *Mi-1.2+*) or susceptible (Moneymaker, *Mi-1.2–*) leaf section (3mm^2^) for a no-choice bioassay (*n*=43), or with both the tissue types for a choice bioassay (*n*=50). Tubes were observed continuously for 1h, and feeding behaviours of the predator were recorded manually using the Etholog software version 2.25 (Ottoni, 2000). The feeding behaviour of *O. insidiosus* on aphid prey was determined using similar choice and no-choice assays, but instead of leaf materials, *O. insidiosus* adults were presented with first instar potato aphids that had been reared on either *Mi-1.2+* or *Mi-1.2–* tomato plants. For predation assays, the predators were starved for ~18h prior to the no-choice (1h observation, *n*=9–11) and the choice bioassay (2h observation, *n*=17). The parameters recorded during the no-choice assays included the time at which the predator first touched the plant or probed the prey item with its stylets, the incidence and duration of this first contact and each subsequent contact, and the incidence and duration of grooming behaviour. The choice assays recorded which of the two plant or prey types was contacted first, as well as the number, incidence, and duration of contacts the predator made with each, and the incidence of aphid mortality. For the no-choice assays, the data were analysed by ANOVA. For the choice assays, all parameters were analysed using Chi-square (χ^2^) tests to determine if the proportion of times a specific plant genotype or plant-fed aphid was preferred by *O. insidiosus* (eg contacted first, contacted longer, etc) varied significantly from the 0.5 value that would be expected if host choice were based purely on random chance. When required, data were transformed using the best Box-Cox.

### Molecular analysis to detect plant feeding

For 36h, *O. insidiosus* adult females were deprived of any plant substrate and were only provided moth eggs and hydrocapsules. Then the insects were caged individually on resistant (Motelle) or susceptible (Moneymaker) plants, or were maintained without a food source for the starved control group. After 16h of exposure, the insects were collected from the plants and flash-frozen for DNA extraction (19 individuals per plant genotype; 8 controls starved for 52h). To detect plant feeding in early instars, immatures were reared on resistant and susceptible tomato leaflets and were collected for analysis upon reaching the third instar (20 samples from resistant plants; 19 from susceptible plants; 6 starved control samples; 2 individuals/sample). DNA was extracted with the QIAamp DNA Mini Kit (Qiagen, Inc., Valencia, CA), and Q-PCR was performed on the DNA extracts using two primer sets: one targeting an actin gene from tomato (GQ339765.1), and another targeting a cytochrome oxidase 1 (Co-1) sequence from *O. insidiosus* (EF467230) (Table 1). Q-PCR was performed using an Mx3000P™ Real-Time PCR System (Stratagene; La Jolla, California) and the Clonetech SYBR®ADVANTAGE® qPCR premix Kit (Clonetech, Mountain View, CA) with the following PCR conditions: 10min at 95 °C, 40 amplification cycles (95 °C for 15 s, 55 °C for 30 s, 72 °C for 30 s, and data acquisition at the end of each cycle), and a final data acquisition step to generate melting curves from 65°C to 95°C every 0.3 °C. For each primer set, the amplification efficiency was calculated using the formula, *E*=10^[–1/Ct slope]^ from data generated on serial dilutions of a set of bulked cDNA standards (Rasmussen, 2001). Relative quantification (*RQ*) values of PCR products were calculated using Pfaffl methodology (Pfaffl, 2001). Data from the tomato actin primers were normalized using the insect Co-1 primers to control for differences in insect sizes, and the PCR products from the actin primers in each experimental sample were quantified relative to the average cycle threshold (Ct) value of the starved control samples.

**Table 1.** Primer sets

Purpose	Target gene	Accession no.	Forward primer 5′–3′	Reverse primer 5′–3′	Size of amplicon from genomic DNA	Size of amplicon from cDNA
Molecular gut	*ß-Actin*	FJ532351.1	GGAAAAGCTTGCC	CCTGCAGCTTCC	180 bp	NA
content analysis	(tomato)		TATGTGG	ATACCAAT		
Molecular gut	Co-I	EF467230	ACACATTATTAGA	TAAATAGAAATA	280 bp	NA
content analysis	(*O. insidiosus*)		AAAGAAAGAGGA	CGAATCCTAATG		
*In situ* RT-PCR	*Mi-1.2*	AF039682.1	GCAAACAACTCAT	ACATCATCATCC	NA	248 bp
	(tomato)		TGGTGTT	AAAAGTGA		
*In situ* RT-PCR	*Prosystemin*	M84801	AACAAAGGAGATG	CCTTTACATATC	NA	200 bp
	(tomato)		ACATGC	CTCCCTCC		
*In situ* RT-PCR	*G2-LTF*	TC118156	CGCTTCGTTGATG	AGCTCTATCGCC	500 bp	186 bp
	(tomato)		CTGTTAC	ATCCTTCA		
*In situ* RT-PCR	*Rpl2*	NC_007898	CATAGAAATCACA	GGGATGGTCTA	265 bp	265 bp
	(tomato)		CTTGGAAAGG	CAGGGTTCA		

The *RQ* values of the samples were log_2_-transformed to stabilize variances and analysed by one-way ANOVA, and means for significant effects were separated using Student’s *t*-tests with JMP^®^ v9.0 (SAS Institute, Cary, NC). To compare the proportion of each treatment group that was positive for plant DNA, samples were classified as positive for plant DNA if their *RQ* value was more than two standard deviations above the average *RQ* value for the starved controls. Then the percentages of positive samples were compared among treatment groups using Chi-square analysis.

### Localization of *Mi-1.2* transcripts

Tissue sections from petioles of Moneymaker (*Mi-1.2–*) and 143-25 (*Mi-1.2+ Tr*) were cryofixed as previously described (Nakazono *et al.*, 2003), embedded in Tissue-Tek® OCT medium (Sakura Finetek USA, Torrance, CA), and cryosectioned at 20 μm and –19 °C using a Leica CM 3050S cryostat (Leica Microsystems, Frisco, Texas). Laser capture microdissection (PALM MicroLaser Systems, Zeiss, Bernried, Germany) was used to collect samples of phloem, epidermal, and mesophyll cells (5×10^6^ μm^2^ area per sample; replicates from three plants per genotype for a total of 18 samples (see Supplementary Fig. S1 at *JXB* online). RNA was extracted from the catapulted tissue sections using the PicoPure™ RNA isolation kit (Arcturus, Sunnyvale, CA) and DNase treated using the RNase-free DNase set kit (Qiagen Inc, Valencia, CA). The Experion RNA HighSens Analysis Kit (Bio-Rad Laboratories, Wilmington, USA) was used to confirm RNA quality and integrity. A single round of amplification and cDNA synthesis were performed on ~1ng RNA per sample using the Arcturus® RiboAmp® HS PLUS 1.5 Round kit (Molecular Devices, Mountain View, CA) and Superscript III (Invitrogen, Carlsbad, CA). PCR was performed on ~80ng of cDNA/sample using primers to amplify *Mi-1.2, Prosystemin, Ribosomal protein L2,* and a G2-like transcription factor homologous to a phloem-specific (Banerjee *et al.*, 2006) mRNA in potato (accession no. TC118156, Gene Index-DFCI) (Table 1). PCR conditions were as follows: 95 °C for 3min, 30 cycles (95 °C for 30 s, 55 °C for 30 s, 72 °C for 60 s), and 72 °C for 5min.

## Results

### Influence of resistance on *O. insidiosus* oviposition, emergence, and survival

**Fig. 1.**
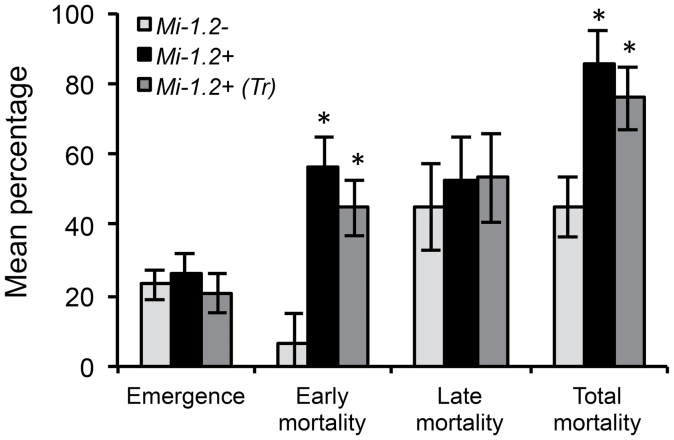
Emergence and mortality of *O. insidiosus* immatures on intact plants with (+) and without (–) the *Mi-1.2* resistance gene. Insect performance was measured in no-choice assays on the tomato cultivar Moneymaker (*Mi-1.2–*), the nearly isogenic cultivar Motelle (*Mi-1.2+*), and 143-25, a transgenic line that expresses *Mi-1.2* in a Moneymaker background [*Mi-1.2+ (Tr)*]. The percentage of emergence (Mean±SEM) was calculated as (number of *O. insidiosus* emerged/number of eggs laid)×100. Early mortality denotes the percentage of emerged immatures that died in the first or second instar, and total mortality denotes the percentage of emerged immatures that died before reaching adulthood. Insect mortality on plant genotypes labelled with asterisks (*) are significantly different from that on the susceptible control line (*Mi-1.2–*) at α=0.05.

To determine whether exposure to resistant plants has any direct effects on *O. insidiosus*, adults were allowed to oviposit on intact tomato plants, and rates of oviposition, emergence, and offspring survival were compared on three genotypes: a tomato cultivar that lacks *Mi-1.2* (cv. Moneymaker, *Mi-1.2–*), a nearly-isogenic line that carries an ~650kb insertion containing *Mi-1.2* (cv. Motelle, *Mi-1.2+*), and a line that expresses *Mi-1.2* transgenically in a Moneymaker background (line 143-25, *Mi-1.2+ Tr*). Although rates of oviposition and offspring emergence were similar on all three genotypes (ANOVA: *P* >0.1), the percentage of offspring that died before reaching adulthood (total mortality; Fig. 1) varied significantly (ANOVA: *P*=0.0063). Total offspring mortality was ~2 times higher on the resistant cultivar Motelle (*Mi-1.2+*) than on the susceptible cultivar Moneymaker (*Mi-1.2–*) (Student’s *t*-test: *P*=0.0025), and ~1.8 times higher on 143-25 (*Mi-1.2+ Tr*) compared with Moneymaker (Student’s *t*-test: *P*=0.0151). Much of the observed mortality occurred in the first and second instars (early mortality; Fig. 1). Early instar mortality varied significantly among treatments (ANOVA: *P* <0.0001), and was ~9 times greater on Motelle (Student’s *t*-test: *P* <0.0001) and ~7 times greater on 143-25 (*P*=0.0001) than on the susceptible plants. Late instar mortality was not significantly different between resistant and susceptible plants (ANOVA: *P* >0.1). Offspring were provisioned with moth eggs from artificial diet-reared laboratory cultures to ensure that the quality of prey provided to the *O. insidiosus* was unaffected by the host plants; therefore, the high *O. insidiosus* mortality observed on genotypes that carry *Mi-1.2* can be attributed to direct interactions between the plant and the predators. The fact that *O. insidiosus* survival on 143-25 was similar to survival on Motelle (Student’s *t*-tests: *P* >0.1) also indicates that the effects of plant genotype on predator survival were due to *Mi-1.2* rather than to other linked genes in the 650kb introgressed region carried by Motelle.

### Influence of resistance on *O. insidiosus* feeding behaviour

**Fig. 2.**
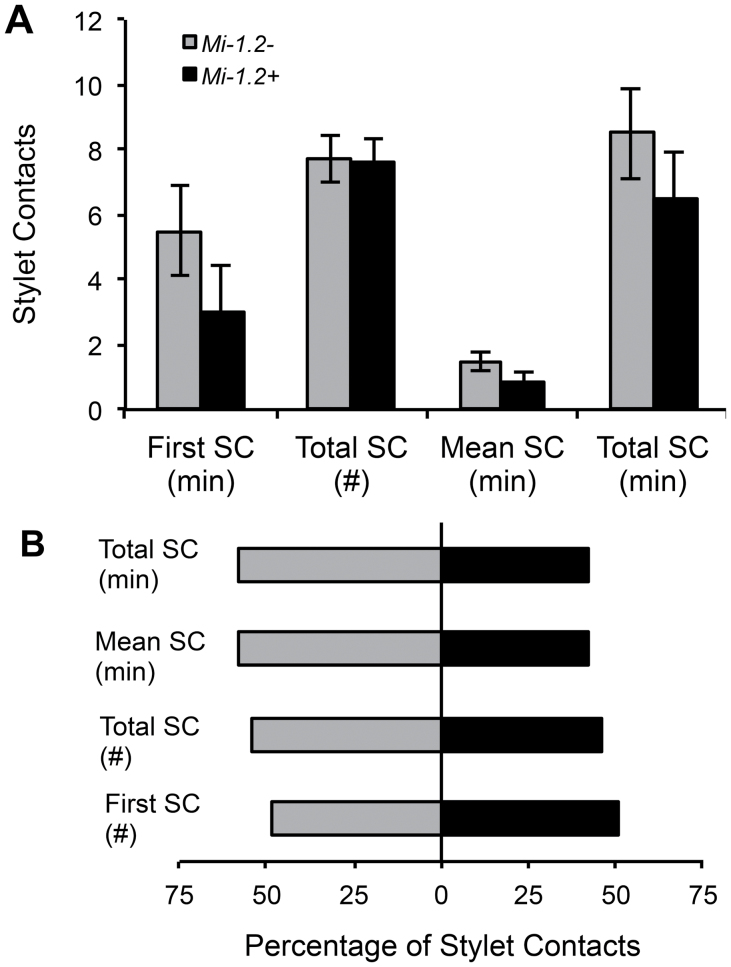
Feeding behaviour of *O. insidiosus* on excised leaf tissue. (A) No-choice assay. Adults of *O. insidiosus* were caged individually with *Mi-1.2–* or *Mi-1.2+* leaf sections (B) Choice assay. Each insect was enclosed with two leaf sections (*Mi-1.2–* and *Mi-1.2+*). Bars represent Mean±SEM. First SC (min)=the time elapsed before the insect first contacted the leaf tissue; first SC (#)=the percentage of insects in the choice assay that contacted the specified genotype before sampling the other genotype; total SC (#)=the total number of stylet contacts per insect; mean SC (min)=the mean duration per stylet contact per insect; and total SC (min)=the total duration of all stylet contacts per insect. (B) The percentage of *O. insidiosus* that had their first SC, their highest number of total SC, or their longest mean SC or total SC duration on *Mi-1.2–* (grey) versus *Mi-1.2+* (black) tissues.

**Fig. 3.**
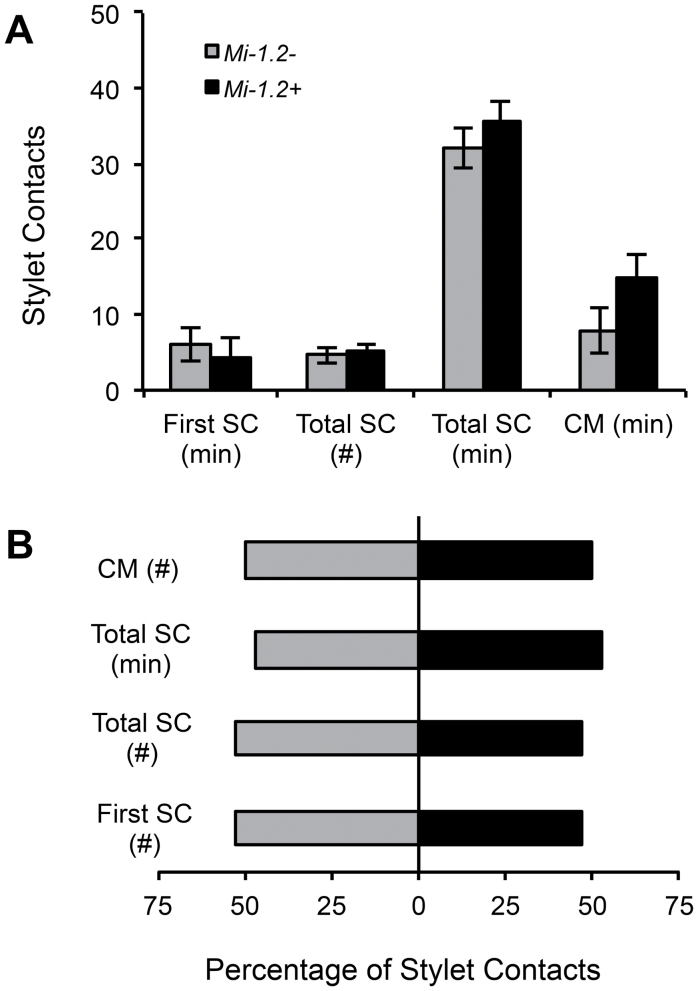
Feeding behaviour of *O. insidiosus* on aphids from *Mi-1.2–* or *Mi-1.2+* tomato plants. (A) No-choice assay. One adult female of *O. insidiosus* was caged with one first instar aphid reared on either a *Mi-1.2–* or a *Mi-1.2+* plant. (B) Choice assay. Each predator was placed in the choice arena with two aphids (1 from *Mi-1.2–* and 1 from *Mi-1.2+* plants). Bars represent Mean±SEM. First SC (min)=the time elapsed before the predator began to stylet contact and probe the aphid; first SC (#)=the percentage of predators in the choice assay that contacted the specified aphid type before sampling the other aphid; total SC (#)=the total number of stylet contacts per predator; total SC (min)=the total duration of all stylet contacts per predator; CM (min)=time to confirmed aphid mortality; and CM(#)=proportion of predators that killed the specific aphid type before other. (B) The proportion of *O. insidiosus* individuals that had their first SC, their highest number of total SC, or their longest total SC duration on aphids from *Mi-1.2–* (grey) versus *Mi-1.2+* (black) tissues.

Behavioural bioassays were conducted to determine if *Mi-1.2* impacts feeding on plants or aphids by *O. insidiosus*. None of the feeding or grooming behavioural parameters that were measured differed significantly between treatments. When *O. insidiosus* were exposed to foliage from resistant and susceptible genotypes in no-choice bioassays, the predators made stylet contact with either plant tissue within ~5min of its introduction into the arena, indicating considerable interaction of the generalist predator with the first trophic level regardless of the plant genotype (Motelle, *Mi-1.2+*; Moneymaker, *Mi-1.2–*). The predator interacted equally with the resistant and susceptible plant genotypes in the no-choice (*n*=43, ANOVAs; *P* >0.1) and choice assays (*n*=50; χ^2^; *P* >0.1; Fig. 2). When *O. insidiosus* were exposed to aphids collected from resistant and susceptible genotypes in no-choice bioassays, the predators started to feed on the aphids within 10min after introduction into the arena and, in most cases, fed longer than 50% of the recording time, regardless of the source of aphids (*Mi-1.2–* or *Mi-1.2+* plant fed). When given a choice between the two types of aphids, *O. insidiosus* prey feeding behaviour and aphid mortality did not suggest any significant preference between the two aphid types (χ^2^ tests: *P* >0.1; Fig. 3). The predator indiscriminately attacked aphids raised on either of the plant genotypes in both the no-choice (*n*=9, 11) and choice assay (*n*=17). In summary, our results indicated that *O. insidiosus* readily contacts tomato genotypes with and without *Mi-1.2*, in addition to accepting aphid prey reared on either genotype (Figs 2, 3).

### Molecular analysis to detect plant feeding

**Fig. 4.**
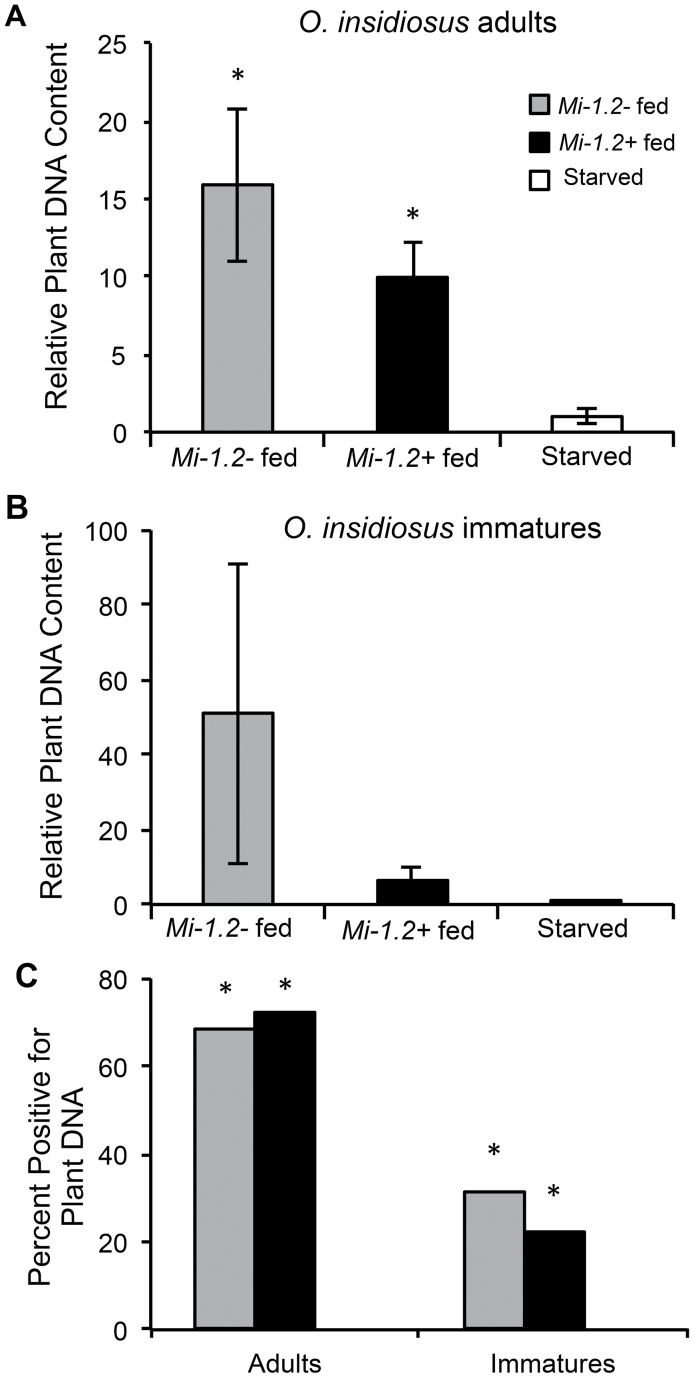
Gut content analysis of *O. insidiosus* to detect plant feeding. Q-PCR was used to detect tomato DNA in *O. insidiosus* adults and immatures after the predators were allowed to feed on resistant (Motelle, *Mi-1.2+*) or susceptible (Moneymaker, *Mi-1.2–*) tomato plants. The starved control group was not fed any plant tissue for the duration of the experiment. The relative quantification (*RQ*) values for plant DNA in adults (A) and immatures (B) were normalized based on amplification of a DNA sequence from *O. insidiosus*, and were expressed relative to the mean cycle threshold (Ct) values for starved controls. (C) Insect samples were categorized as positive for plant DNA if their *RQ* values were greater than two standard deviations above the mean *RQ* value for the starved controls. Treatments labelled with asterisks (*) were significantly different from the corresponding starved control group at α=0.05.

Real-time PCR was used to detect tomato DNA in *O. insidiosus* adults and immatures exposed to resistant and susceptible plants in order to detect plant feeding and estimate its frequency on these two genotypes. Estimates of the abundance of tomato DNA in insect samples was normalized to estimates of insect DNA quantities to correct for possible variation in insect sizes. Relative quantification (*RQ*) values for tomato DNA were then calculated for each sample relative to the mean values for the negative controls (*O. insidiosus* that were deprived of plant materials for the duration of the experiment). For adults, there was a significant difference (ANOVA: *P*=0.0011) in the relative quantity of plant DNA present among the treatment groups (*Mi-1.2+*, *Mi-1.2–*, and starved), with significantly higher *RQ* values for insects that were exposed to resistant (Student’s *t*-test: *P*=0.0003) or susceptible plants (Student’s *t*-test: *P*=0.0031) compared with the starved controls (Fig. 4A). The average relative quantity of plant DNA in adults from susceptible plants was 1.6 times higher than the DNA content from resistant plants, but this difference was not statistically significant (Student’s *t*-test: *P*=0.229). For immature *O. insidiosus*, there was no significant difference in *RQ* values among the three treatment groups (ANOVA: *P*=0.3487), possibly because the relative quantities of plant DNA were highly variable (Fig. 4B). To explore variation that may have arisen from insects that did not feed, the proportion within each treatment group that was positive for plant DNA (i.e. those with *RQ* values more than two standard deviations above the average *RQ* value for the starved controls) was also examined. Compared with the starved controls which did not generate any samples above the *RQ* threshold, all other treatment groups had a significantly higher number of positive samples (Fig. 4C; Effect likelihood ratio tests: *P* <0.05), indicating that feeding occurred in all four treatment groups (adults and immatures exposed to *Mi-1.2+* and *Mi-1.2–* plants). Feeding appeared to be more common among adults (68–72%) than immatures (22–32%).

### Localization of *Mi-1.2* transcripts

**Fig. 5.**
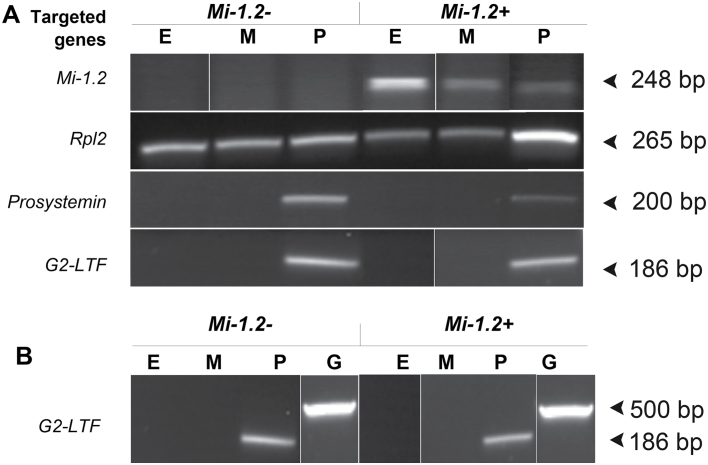
Localization of *Mi-1.2* transcripts in tomato tissues. (A) RT-PCR was used to assess expression of *Mi-1.2* in epidermis (E), mesophyll (M), and phloem (P) samples harvested by LCM from the petioles of 143-25 (*Mi-1.2*+) and the susceptible cultivar Moneymaker (*Mi-1.2–*). Primers to the ubiquitously-expressed housekeeping gene *Ribosomal protein L2* (*Rpl2*) were used to confirm good cDNA synthesis, and primers that amplify phloem-specific transcripts (*Prosystemin* and *G2-like transcription factor*) were used to confirm that the microdissection procedure adequately separated vascular and non-vascular tissues. (B) The *G2-like transcription factor* (G2-LTF) primers were also used to confirm the absence of genomic DNA in the samples, because the primers flank an intron and generate different amplicon sizes from genomic DNA (G) versus our cDNA templates. The gel lanes in this figure were spliced and rearranged to conveniently compare gene expression among the targeted tissues.

*Orius insidiosus* does not feed from the phloem like other pests that are impacted by *Mi-1.2*. To detect low levels of *Mi-1.2* expression and to discriminate this gene from its many close homologues in tomato, a combination of laser capture microdissection (LCM) and RT-PCR were used to determine the localization of *Mi-1.2* transcripts in resistant plant tissues. Cryosectioning and LCM were used to dissect samples of epidermal, mesophyll, and phloem tissues from tomato plants that lack *Mi-1.2* (cv. Moneymaker), and isogenic transgenic plants (143-25) that express *Mi-1.2* driven by its native promoter in the same genetic background (cv. Moneymaker). RT-PCR performed on RNA from the harvested tissue provided evidence that the *Mi-1.2* gene is transcribed in all three tissue types (Fig. 5A). The experiment was repeated three times using separate plants. In 143-25, *Mi-1.2* transcripts were detected in all three samples of epidermis tissue, two out of three samples of mesophyll tissue, and one out of three samples of phloem tissue. Primers to detect *Mi-1.2* expression did not generate PCR products from any of the tissue samples from susceptible plants, indicating that our primers did not amplify any of the *Mi-1* homologues present in this genetic background. The presence of cDNA in all samples was confirmed by amplifying transcripts encoding the ribosomal protein RPL2 (Fig. 5A). Primers for *prosystemin* and a G2-like transcription factor (*G2-LTF*), which are expressed specifically in the phloem, detected transcripts in the phloem samples but not in any of the epidermis or mesophyll samples, providing evidence that the LCM sectioning adequately isolated the tissue types (Fig. 5A). The *prosystemin* primers generated PCR products in two out of the three phloem samples, while G2-LTF expression was detected in all three phloem samples. Results for the G2-LTF primers also indicated that our samples were free of contamination from genomic DNA. This primer set flanks an intron and generates a PCR product of ~500bp from genomic DNA samples, whereas it only yields a single amplicon of 186bp from our cDNA samples (Fig. 5B).

## Discussion

The objective of our study was to evaluate the influence of R-gene-mediated resistance in tomato on a common insect predator that feeds supplementally from host plants. Our results showed for the first time that *Mi*-mediated resistance in tomato could have direct non-target effects on a biological control agent. Early instars of the zoophytophagous predator *O. insidiosus* had significantly reduced survival on plants with *Mi-1.2* compared with isogenic susceptible controls (*Mi-1.2–*), even when the predators were provisioned with moth eggs whose quality was unaffected by the host plant. In behavioural assays, resistance did not impact rates of plant sampling by adult predators (Fig. 2); moreover, molecular gut content analysis confirmed that adult and immature *O. insidiosus* fed on both resistant and susceptible plant genotypes. These data suggest that the deleterious effects of resistant plants on *O. insidiosus* were due to antibiosis rather than antixenosis. However, in our molecular gut content analysis, there was a trend for lower levels of plant contents in insects exposed to resistant plants compared with insects on susceptible genotypes. Therefore, non-preference may also contribute to the effects of *Mi*-mediated resistance on *O. insidiosus*. Further, it is possible that our behavioural studies with adult *O. insidiousus* may not reflect the behaviour of all developmental stages; although early-instar nymphs are the life stage most impacted by *Mi-1.2*, behavioural assays with this life stage were not performed because they are very delicate and are prone to injury when handled.

*Orius insidiosus* is known to feed on xylem sap, pollen, and cell contents from epidermal or mesophyll layers, but monitoring of radiolabelled tracers indicates that it does not ingest phloem sap (Armer *et al.*, 1998). Thus, *O. insidiosus* is the first organism to be impacted by *Mi-1.2* that falls outside the feeding guild of herbivores that specialize on phloem sap. Consistent with this observation, it has been confirmed that the *Mi-1.2* gene is expressed in the epidermis and mesophyll in addition to the vasculature. Furthermore, recent electrophysiological observations of aphid and whitefly feeding behaviour on plants with *Mi-1.2* suggest that these insects are also impacted by deterrents located outside the phloem. The slender, tubular mouthparts of aphids and whiteflies penetrate between the cells of the epidermis and mesophyll, but will intermittently sample these cells en route to the phloem. Electrical penetration graph recordings of aphids and whiteflies on plants with and without *Mi-1.2* indicate that this sampling behaviour is delayed on resistant varieties, suggesting that these insects may encounter deterrents that act in the intercellular spaces of the epidermis or mesophyll (Jiang *et al.*, 2001; Pallipparambil *et al.*, 2010). Together, these observations suggest that *Mi*-mediated resistance involves recognition events and defence responses that occur in the epidermis or mesophyll, rather than depending solely upon phloem-limited defences. Moreover, our observations indicate that *Mi-1.2* can impact a greater ecological diversity of organisms than previously assumed.

The vast majority of previous studies on the non-target impacts of herbivore resistance in plants have focused on transgenic traits such as the expression of Cry toxins from *Bacillus thuringiensis* Berliner, because there are regulatory requirements for assessing the risks of genetically modified crops (Lundgren *et al.*, 2009*a*; Gatehouse *et al.*, 2011). Fewer studies have examined the potential non-target effects of resistant crop varieties developed through traditional breeding and, of these, only a handful have explored the impacts of R genes, which have typically been assumed to have very narrow target ranges. In rice, alfalfa, and wheat, R genes conferring aphid or planthopper resistance have been reported to be compatible with biological control, with no negative effects detected on predators or parasitoids (Salim and Heinrichs, 1986; Farid *et al*., 1997, 1998*a*, *b*; Messina and Sorenson, 2001; Bosque-Pérez *et al.*, 2002; Giles *et al.*, 2002). However, in raspberry, lower parasitoid attack rates were observed on plants carrying the A_10_ aphid resistance gene than on susceptible checks, possibly due to lower aphid densities and the increased incidence of aphids dropping off the resistant plants (Mitchell *et al.*, 2010). Moreover, in soybean, the *Rag1* aphid resistance gene appears to have direct negative effects on the zoophytophagous predator *Harmonia axyridis* (Pallas), and also reduces attack rates and adult emergence of *Binodoxys communis* (Gahan), a parasitoid of aphids (Lundgren *et al.*, 2009*b*; Chacón *et al.*, 2012; Ghising *et al.*, 2012). These results demonstrate that non-target effects are not limited to genetically modified crops, and that even R gene-mediated resistance can directly impact the third trophic level.

Our findings also indicate that there is a need to characterize plant responses to *O. insidiosus* and other zoophytophagous predators. The fact that the *Mi-1.2* R gene impacts *O. insidiosus* suggests that effectors from this insect elicit a defence response in the host plant. Because omnivorous Heteropterans such as *O. insidiosus* utilize extra-oral digestion, they inject enzyme-rich saliva into their hosts and these secretions could trigger plant defences or enhance the suitability of susceptible hosts similar to oral secretions from caterpillars, grasshoppers, aphids, and other herbivores. Moreover, research with other herbivores has revealed that plant defence responses can be triggered by a variety of other cues from insects, including oviposition damage, egg deposition, and even walking (Peiffer *et al.*, 2009). Therefore, the presence of omnivores and other natural enemies on plants could modify the suitability of host plants for both zoophytophagous species and herbivores. For example, salivary extracts from the omnivorous species *Lygus hesperus* Knight alter plant volatile emissions (Rodriguez-Saona *et al.*, 2002), feeding by *Tupiocoris notatus* (Distant) modifies photosynthetic rates (Halitschke *et al.*, 2011), and oviposition by *O. laevigatus* (Fieber) induces wound-responsive defences that decrease infestations by the Western flower thrips *Frankliniella occidentalis* (Pergande) (De Puysseleyr *et al.*, 2011). Mostly though, little is known about the salivary excretions of *O. insidiosus* and other zoophytophagous species, or about their impact on induced defences in host plants. Further research is required to understand and predict their tritrophic interactions with plants and herbivores.

Field studies are also needed to assess the overall impact of *Mi-1.2* on biological control agents in tomato. While controlled studies in the laboratory, growth chamber, or greenhouse have the greatest power to detect negative impacts of host plant resistance on natural enemies and to decipher the mode of action of these effects, field experiments have greater power to evaluate the real-world consequences of these effects for pest management (Romeis *et al.*, 2011). The net effect of *Mi*-mediated resistance on *O. insidiosus* predation rates under field conditions will depend in part upon (i) the effects of resistance on prey abundance, densities, spatial distributions, size and quality; (ii) the potential for *O. insidiosus* to compensate for low-quality host plants by increasing their rate of prey consumption; and (iii) their ability to escape resistance in time or space by feeding on tissues in which *Mi*-mediated aphid resistance is not active, such as floral apexes (Kaloshian *et al.*, 1995). Furthermore, the net effect of *Mi-1.2* on pest populations under field conditions will depend upon the relative weight of bottom-up versus top-down effects on herbivores. For example, resistant soybean cultivars provided improved management of the Mexican bean beetle *Epilachna varivestis* Mulsant despite the fact that they reduced attack rates by the predator *Podisus maculiventris* (Say) (Bartlett, 2008). Similarly, although resistant varieties of *Vicia faba* L. appeared to increase the development time and reduce the fecundity of the predator *Coccinella septempunctata* L., aphid numbers were lowest when the two management strategies were combined (Shannag and Obeidat, 2008). In fact, many studies report that biological control is most likely to achieve economically acceptable results when deployed in combination with a crop variety that is at least partially resistant to the target pest (Van Emden, 1995). Indeed, supplemental control strategies such as biological control are particularly important for crops in which only partial resistance is available. In the case of tomato cultivars that carry the *Mi-1.2* gene, biotypes of the potato aphid have been identified that are partially or fully tolerant to resistance (Rossi *et al.*, 1998; Hebert *et al.*, 2007), and so supplemental control strategies are needed to manage these virulent or semi-virulent populations. Moreover, *O. insidiosus* preys on mites, thrips, and other pests that are not managed by *Mi*-mediated resistance. In short, any cost–benefit analysis of host plant resistance must take into consideration the full suite of pests and beneficial insects that are present, and measure the independent and combined effects of resistance and biological control agents on pest populations.

## Supplementary data

Supplementary data can be found at *JXB* online.

Supplementary Fig. S1. Micrographs obtained from PALM RoboSoftware show dissection of (A) phloem tissue, and (B) mesophyll and epidermis from cryosections of tomato leaf petioles using the Laser Capture Microdissection (LCM) technique.

Supplementary Data

## References

[CIT0001] Armer CA Wiedenmann RN Bush DR 1998 Plant feeding site selection on soybean by the facultatively phytophagous predator *Orius insidiosus* . Entomologia Experimentalis et Applicata 86, 109–118.

[CIT0002] Banerjee AK Chatterjee M Yu Y Suh S-G Miller WA Hannapel DJ 2006 Dynamics of a mobile RNA of potato involved in a long-distance signaling pathway. The Plant Cell Online 18, 3443–3457. 10.1105/tpc.106.042473 PMC1785412 17189340

[CIT0003] Bartlett R 2008 Negative interactions between chemical resistance and predators affect fitness in soybeans. Ecological Entomology 33, 673–678.

[CIT0004] Bosque-Pérez NA Johnson JB Schotzko DJ Unger L 2002 Species diversity, abundance, and phenology of aphid natural enemies on spring wheats resistant and susceptible to Russian wheat aphid. BioControl 47, 667–684.

[CIT0005] Casteel C Walling LL Paine T 2006 Behavior and biology of the tomato psyllid, *Bactericerca cockerelli*, in response to the *Mi-1.2* gene. Entomologia Experimentalis et Applicata 121, 67–72.

[CIT0006] Chacón JM Asplen MK Heimpel GE 2012 Combined effects of host-plant resistance and intraguild predation on the soybean aphid parasitoid *Binodoxys communis* in the field. Biological Control 60, 16–25.

[CIT0007] Coll M 1996 Feeding and ovipositing on plants by an omnivorous insect predator. Oecologia 105, 214–220. 10.1007/BF00328549 28307085

[CIT0008] Cooper WC Jia L Goggin FL 2004 Acquired and R-gene-mediated resistance against the potato aphid in tomato. Journal of Chemical Ecology 30, 2527–2542. 15724969 10.1007/s10886-004-7948-9

[CIT0009] De Puysseleyr V Höfte M De Clercq P 2011 Ovipositing *Orius laevigatus* increase tomato resistance against *Frankliniella occidentalis* feeding by inducing the wound response. Arthropod-Plant Interactions 5, 71–80.

[CIT0010] Dropkin VH 1969 Cellular responses of plants to nematode infections. Annual Review of Phytopathology 7, 101–122.

[CIT0011] Eigenbrode SD 2001 Attachment by predatory insects to waxy plant surfaces: Mechanisms and ecological implications. American Zoologist 41, 1435–1436.

[CIT0012] Eubanks MD Styrsky JD 2005 Effects of plant feeding on the performance of omnivorous ‘predators’. In: Wackers FL van Rijn PCJ Bruin J, eds. Plant-provided food for carnivorous insects: a protective mutualism and its applications. New York: Cambridge University Press, 148–177.

[CIT0013] Farid A Johnson JB Quisenberry SS 1997 Compatibility of a coccinellid predator with a Russian wheat aphid resistant wheat. Journal of the Kansas Entomological Society 70, 114–119.

[CIT0014] Farid A Johnson JB Shafii B Quisenberry SS 1998a Tritrophic studies of Russian wheat aphid, a parasitoid, and resistant and susceptible wheat over three parasitoid generations. Biological Control 12, 1–6.

[CIT0015] Farid A Quisenberry SS Johnson JB Shafii B 1998b Impact of wheat resistance on Russian wheat aphid and a parasitoid. Journal of Economic Entomology 91, 334–339.

[CIT0016] Flor HH 1955 Host–parasite interaction in flax rust—its genetics and other implications. Phytopathology 45, 680–685.

[CIT0017] Gatehouse A Ferry N Edwards M Bell H 2011 Insect-resistant biotech crops and their impacts on beneficial arthropods. Philosophical Transactions of the Royal Society B: Biological Sciences 366, 1438–1452. 10.1098/rstb.2010.0330 PMC3081576 21444317

[CIT0018] Ghising K Harmon JP Beauzay PB Prischmann-Voldseth DA Helms TC Ode PJ Knodel JJ 2012 Impact of *Rag1* aphid resistant soybeans on *Binodoxys communis* (Hymenoptera: Braconidae), a parasitoid of soybean aphid (Hemiptera: Aphididae). Environmental Entomology 41, 282–288. 22507000 10.1603/EN11196

[CIT0019] Giles KL Berberet RC Zarrabi AA Dillwith JW 2002 Influence of alfalfa cultivar on suitability of *Acyrthosiphon kondoi* (Homoptera: Aphididae) for survival and development of *Hippodamia convergens* and *Coccinella septempunctata* (Coleoptera: Coccinellidae). Journal of Economic Entomology 95, 552–557. 12075999 10.1603/0022-0493-95.3.552

[CIT0020] Goggin FL Shah G Williamson VM Ullman DE 2004 Developmental regulation of *Mi*-mediated aphid resistance is independent of Mi-1.2 transcript levels. Molecular Plant–-Microbe Interactions 17, 532–536. 15141957 10.1094/MPMI.2004.17.5.532

[CIT0021] Goggin FL Williamson VM Ullman DE 2001 Variability in the response of *Macrosiphum euphorbiae* and *Myzus persicae* (Hemiptera: Aphididae) to the tomato resistance gene *Mi* . Environmental Entomology 30, 101–106.

[CIT0022] Halitschke R Hamilton JG Kessler A 2011 Herbivore‐specific elicitation of photosynthesis by mirid bug salivary secretions in the wild tobacco *Nicotiana attenuata* . New Phytologist 191, 528–535. 21443673 10.1111/j.1469-8137.2011.03701.x

[CIT0023] Hebert SL Jia LL Goggin FL 2007 Quantitative differences in aphid virulence and foliar symptom development on tomato plants carrying the *Mi* resistance gene. Environmental Entomology 36, 458–467. 17445382 10.1603/0046-225x(2007)36[458:qdiava]2.0.co;2

[CIT0024] Jiang YX Nombela G Muniz M 2001 Analysis by DC-EPG of the resistance to *Bemisia tabaci* on an *Mi*-tomato line. Entomologia Experimentalis et Applicata 99, 295–302.

[CIT0025] Kaloshian I Kinsey MG Williamson VM Ullman DE 2000 *Mi*-mediated resistance against the potato aphid *Macrosiphum euphorbiae* (Hemiptera: Aphididae) limits sieve element ingestion. Environmental Entomology 29, 690–695.

[CIT0026] Kaloshian I Lange WH Williamson VM 1995 An aphid-resistance locus is tightly linked to the nematode-resistance gene, *Mi*, in tomato. Proceedings of the National Academy of Sciences, USA 92, 622–625. 10.1073/pnas.92.2.622 PMC42794 11607509

[CIT0027] Kennedy GG 2003 Tomato, pests, parasitoids, and predators: tritrophic interactions involving the genus *Lycopersicon* . Annual Review of Entomology 48, 51–72. 10.1146/annurev.ento.48.091801.112733 12194909

[CIT0028] Lozano-Torres JL Wilbers RHP Gawronski P Boshoven JC Finkers-Tomczak A Cordewener JHG America AHP Overmars HA Van’t Klooster JW Baranowski L 2012 Dual disease resistance mediated by the immune receptor Cf-2 in tomato requires a common virulence target of a fungus and a nematode. Proceedings of the National Academy of Sciences, USA 109, 10119–10124. 10.1073/pnas.1202867109 PMC3382537 22675118

[CIT0029] Lundgren JG Fergen JK Riedell WE 2008 The influence of plant anatomy on oviposition and reproductive success of the omnivorous bug *Orius insidiosus* . Animal Behaviour 75, 1495–1502.

[CIT0030] Lundgren JG Gassmann AJ Bernal J Duan JJ Ruberson J 2009a Ecological compatibility of GM crops and biological control. Crop Protection 28, 1017–1030.

[CIT0031] Lundgren JG Hesler LS Tilmon K Dashiell K Scott R 2009b Direct effects of soybean varietal selection and *Aphis glycines*-resistant soybeans on natural enemies. Arthropod-Plant Interactions 3, 9–16.

[CIT0032] Messina FJ Sorenson SM 2001 Effectiveness of lacewing larvae in reducing Russian wheat aphid populations on susceptible and resistant wheat. Biological Control 21, 19–26.

[CIT0033] Milligan SB Bodeau J Yaghoobi J Kaloshian I Zabel P Williamson VM 1998 The root knot nematode resistance gene *Mi* from tomato is a member of the leucine zipper, nucleotide binding, leucine-rich repeat family of plant genes. The Plant Cell 10, 1307–1319. 9707531 10.1105/tpc.10.8.1307 PMC144378

[CIT0034] Mitchell C Johnson S Gordon S Birch A Hubbard S 2010 Combining plant resistance and a natural enemy to control *Amphorophora idaei* . BioControl 55, 321–327.

[CIT0035] Nakazono M Qiu F Borsuk LA Schnable PS 2003 Laser-capture microdissection, a tool for the global analysis of gene expression in specific plant cell types: identification of genes expressed differentially in epidermal cells or vascular tissues of maize. The Plant Cell Online 15, 583–596. 10.1105/tpc.008102 PMC150015 12615934

[CIT0036] Nombela G Beitia F Muniz M 2001 A differential interaction study of *Bemisia tabaci* Q-biotype on commercial tomato varieties with or without the *Mi* resistance gene, and comparative host responses with the B-biotype. Entomologia Experimentalis et Applicata 98, 339–344.

[CIT0037] Obrycki JJ 1986 The influence of foliar pubescence on entomophagous species. In: Boethel DJ Eikenbary RD, eds. *Interactions of plant resistance and parasitoids and predators of insects*. New York, USA: Ellis Horwood, 61–83.

[CIT0038] Ode PJ 2006 Plant chemistry and natural enemy fitness: effects on herbivore and natural enemy interactions. Annual Review of Entomology 51, 163–185. 10.1146/annurev.ento.51.110104.151110 16332208

[CIT0039] Ottoni EB 2000 EthoLog 2.2: a tool for the transcription and timing of behavior observation sessions. Behavior Research Methods Instruments & Computers 32, 446–449. 10.3758/bf03200814 11029818

[CIT0040] Pallipparambil GR Reese JC Avila CA Louis JM Goggin FL 2010 *Mi*-mediated aphid resistance in tomato: tissue localization and impact on the feeding behavior of two potato aphid clones with differing levels of virulence. Entomologia Experimentalis et Applicata 135, 295–307.

[CIT0041] Peiffer M Tooker JF Luthe DS Felton GW 2009 Plants on early alert: glandular trichomes as sensors for insect herbivores. New Phytologist 184, 644–656. 19703113 10.1111/j.1469-8137.2009.03002.x

[CIT0042] Pfaffl MW 2001 A new mathematical model for relative quantification in real-time RT-PCR. Nucleic Acids Research 29, e45. 11328886 10.1093/nar/29.9.e45 PMC55695

[CIT0043] Rasmussen R 2001 Quantification on the LightCycler. In: Meuer S Wittwer C Nakagawara K, eds. Rapid cycle real-time PCR, methods and applications. Heidelberg: Springer Press, 21–34.

[CIT0044] Rodriguez-Saona C Crafts-Brandner SJ Williams III L Paré PW 2002 *Lygus hesperus* feeding and salivary gland extracts induce volatile emissions in plants. Journal of Chemical Ecology 28, 1733–1747. 12449502 10.1023/a:1020552932566

[CIT0045] Romeis J Hellmich RL Candolfi MP 2011 Recommendations for the design of laboratory studies on non-target arthropods for risk assessment of genetically engineered plants. Transgenic Research 20, 1–22. 20938806 10.1007/s11248-010-9446-x PMC3018611

[CIT0046] Rossi M Goggin FL Milligan SB Kaloshian I Ullman DE Williamson VM 1998 The nematode resistance gene *Mi* of tomato confers resistance against the potato aphid. Proceedings of the National Academy of Sciences, USA 95, 9750–9754. 10.1073/pnas.95.17.9750 PMC21408 9707547

[CIT0047] Salim M Heinrichs EA 1986 Impact of varietal resistance in rice and predation on the mortality of *Sogatella furcifera* (Horvath) (Homoptera, Delphacidae). Crop Protection 5, 395–399.

[CIT0048] Shannag HK Obeidat WM 2008 Interaction between plant resistance and predation of *Aphis fabae* (Homoptera: Aphididae) by *Coccinella septempunctata* (Coleoptera: Coccinellidae). Annals of Applied Biology 152, 331–337.

[CIT0049] Smith CM Clement SL 2012 Molecular bases of plant resistance to arthropods. Annual Review of Entomology 57, 309–328. 10.1146/annurev-ento-120710-100642 21910639

[CIT0050] Van Emden HF 1995 Host-plant aphidophaga interactions. Agriculture Ecosystems and Environment 52, 3–11.

[CIT0051] van Ooijen G van den Burg HA Cornelissen BJ Takken FL 2007 Structure and function of resistance proteins in solanaceous plants. Annual Review of Phytopathology 45, 43–72. 10.1146/annurev.phyto.45.062806.094430 17367271

