# Supplementary material for: Mi-1.2, an R gene for aphid resistance in tomato, has direct negative effects on a zoophytophagous biocontrol agent, Orius insidiosus
Source: J Exp Bot. 2014 Sep 4;66(2):549–57. doi: 10.1093/jxb/eru361 (PMC4286404; doi:10.1093/jxb/eru361)
Supplement: Supplementary Data [file supp_66_2_549__index.html]

 Mi-1.2, an R gene for aphid resistance in tomato, has direct negative effects on a zoophytophagous biocontrol agent, Orius insidiosus — Mi-1.2, an R gene for aphid resistance in tomato, has direct negative effects on a zoophytophagous biocontrol agent, Orius insidiosus — Supplementary Data 

# *Mi-1.2*, an R gene for aphid resistance in tomato, has direct negative effects on a zoophytophagous biocontrol agent, *Orius insidiosus*

## Supplementary Data

Data files

**Files in this Data Supplement:**

- Supplementary Data - Supplementary Data
