# Supplementary material for: Mi-1.2, an R gene for aphid resistance in tomato, has direct negative effects on a zoophytophagous biocontrol agent, Orius insidiosus
Source: J Exp Bot. 2014 Sep 4;66(2):549–57. doi: 10.1093/jxb/eru361 (PMC4286404; doi:10.1093/jxb/eru361)
Supplement: Supplementary Data [file supp_eru361_jexbot125955_file001.pdf]

Mi-1.2, an R gene for aphid resistance in tomato, has direct negative effects on a zoophytophagous biocontrol agent, *Orius insidiosus*

Godshen R Pallipparambil, Ronald J Sayler, Jeffrey P Shapiro, Jean MG Thomas, Timothy J Kring, and Fiona L Goggin

Supplementary Data

Fig. S1. Laser Capture Microdissection (LCM). Micrographs obtained from PALM RoboSoftware show dissection of (A) phloem tissue, and (B) mesophyll and epidermis from cryosections of tomato leaf petioles using the LCM technique. Lines represent the boundaries of individual dissected tissue sections.

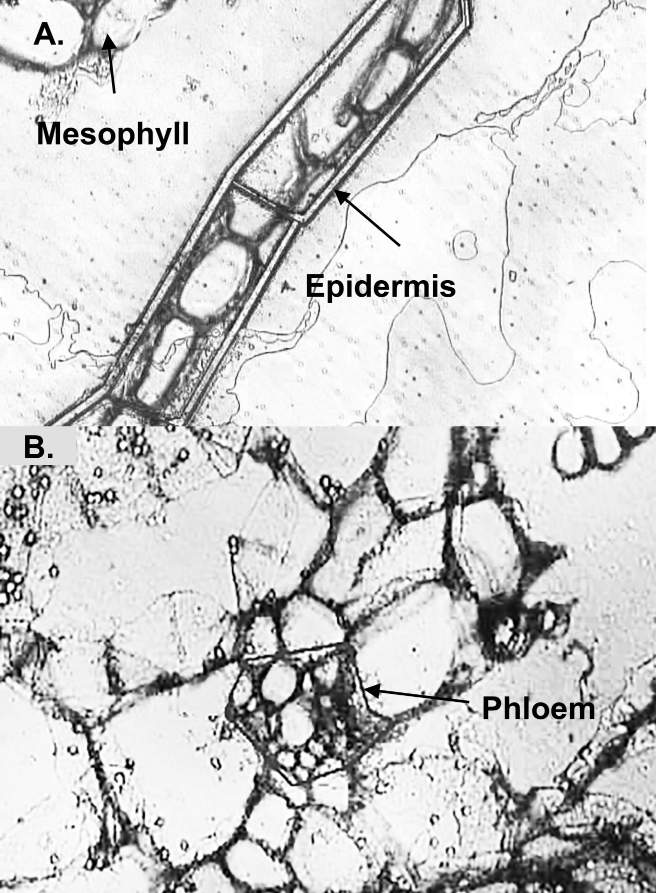
A.
Mesophyll
Epidermis
B.
Phloem
